# Observations on the Febrile Movement in Disease

**Published:** 1858-06

**Authors:** J. R. Hayes


ARTICLE II.

### OBSERVATIONS ON THE *FEBRILE MOVEMENT* IN DISEASE.

(An Essay read before the Rock Island Co. Medical Society, and published at their requst, April 14th, 1858.)

BY J. R. HAYES, M. D.

Mr. President and Gentlemen,—In obedience to your request, I appear before you to-day to offer you a few brief remarks upon a subject of much vital importance, and worthy of our earnest consideration.

It is not my purpose to advance any new fact, doctrine, or theory, in whatever I shall have to say; for, while it may be deemed arrogance on my part, at such an early day of my practical life as a physician, I do not deem myself adequately experienced to enlighten you whatever upon these subjects; but, at the same time, it is a fact that cannot be denied, that every one who has taken up arms in medicine will have, and does have, a theory, peculiar to himself it may be, founded upon facts either apparent or assumed that may have come under his notice. And may I not be tolerated in the remark, that he who never varies from the established rules laid down in our text books, but under all circumstances, right or wrong (and cognizant only of being right by being a faithful copyist), pursues an undeviating track, will sooner or later find himself unsuccessful, if not wholly dissatisfied and disgusted with his profession.

Established fundamental principles and text books we cannot dispense with, nor would we if we could, for it is to them that we are indebted for the accumulated knowledge and experience of years and ages gone by; but these will not suffice if the mind be not schooled to reason over these fields of knowledge, and to apply that knowledge, licensed to that extent to which the judgment may safely and wisely lead.

The most important class of diseases to which the attention and services of a physician are called in this section of the country, is, undoubtedly, that in which the *febrile movement*, as it is termed, predominates, and which seems to be the great evil to be remedied or removed, when called upon to intercede between health and disease.

In relation to the theory of fever, we know that the greatest diversity of opinion has always prevailed. Dr. Stokes tells us, in writing upon fevers generally, that we have not made a single step of advancement since the days of Hippocrates. The *seven* books of Hippocrates contains more in relation to fevers than has since been written upon the subject. In his time, the same protean kinds of fever prevailed as do at this day. Fever, fire, and heat, were synonymous terms to denote the phenomenon of an augmentation of animal temperature, in fact, fever was defined to be nothing more or less than an increase of animal heat. But the cause of fevers is conceded by nearly all modern authors, to be better known at the present time, than in the days of Hippocrates. The origin and effect of malarious influences, are now fully understood, so that it must be conceded (Dr. Stokes to the contrary, notwithstanding), that the moderns have made some advancement in relation to fevers.

It is now fully proved that the proximate cause, is some agent from within or without, acting upon the blood as a poison; this, in turn, acting upon the nerve, centers to the whole nervous system; thence, in all its varied phenomena, to all the other functions in a greater or less degree, in proportion to the severity due the exciting cause—in brief terms, a disease of the whole system.

The blood being therefore the starting point in the inception of fever, may I not be allowed to call to mind a few physiological and pathological facts in relation to the *anatomy* of this fluid, which may be necessary to be borne in mind when treating our fever patients.

I shall call attention to but two constituents of the blood—the fibrin and corpuscles—these being more legitimately connected in their bearing upon each other in inflammatory and febrile affections.

The healthy range of fibrin, as you know, is from *two* to *three* parts in a *thousand*. This proportion is very perceptibly increased during acute inflammatory affections, the increase being strictly proportional to the intensity of inflammation, and fever accompanying the inflammation. The greatest increase is exhibited in pneumonia and rheumatism, and when these diseases alternate to better and worse, the same changes are noticed in the increase and decrease of fibrin. But the increase of fibrin is not due to the febrile condition of itself, but depends upon the amount of inflammation. This is proven by the fact, that in *idiopathic* fevers, the fibrin is diminished, instead of undergoing an increase. In the forming stage of typhoid, it is sometimes reduced so low, as *one* part in a thousand, and the same is true in reference to all fevers arising from *malaria;* but as soon as a local inflammation is developed in the course of the fever, the amount of fibrin runs np to its diseased standard. Another very important fact is, that the amount of fibrin is not diminished by general or local depletion, but on the contrary, has a tendency to increase the amount in proportion as depletion, *general* or *local*, draws upon the more solid constituents of the blood. Abstinence from food, or a continued insufficient supply of it, will very perceptibly *increase* the amount of fibrin, whilst a reduction takes place in the more solid constituents of the blood. *Abstinence*, *depletion*, and *symptomatic fevers*, we thus see, are so closely allied in their effects upon the blood, that the difference is not perceptible. Again, in *idiopathic* fevers, the condition of the fibrin in the blood is the reverse of what it is in *symptomatic* fevers; but *abstinence* and *depletion* will convert it to an analogous condition, as exhibited in symptomatic fevers. How very important is it for us to bear these facts in mind, when making up the diagnosis of our fever patients. In abstinence and depletion, we have *two* powerful agents at our command, the judicious use of which is so necessary to our own success and the salvation of our patients.

It is only a few years since, in the treatment of fevers, that *venesection*, *purgation*, and *starvation*, were practised indiscriminately upon old and young, the feeble and strong, regardless of the temperaments and pathological condition of the unfortunate sick. It is needless to say, that many, very many, must have fallen victims under this *tripod* of surplus interference and medication, wielded by the dogmatic routinists of the day.

I cease to wonder sometimes, that as the plainer truths of physiology began to be partially understood by the masses, they became the ready disciples of empiricism, promulgated by men of but little more information, or knowing better, for mercenary or other selfish motives.

In relation to the corpuscles, we know that the proportion bearing upon the other constituents of the blood varies in different individuals. Take, for example, a patient of a *robust* constitution and one the *antithesis* of this—or one at any point between these two extremes—what invariable rule is there for us to be guided by except it be our reason, *schooled* and *experienced* by the instructions that science, in all its multiplied forms, has afforded us. In every disease of a depressing character, the standard of corpuscles in the blood is lowered very often to an almost infinitesimal amount, or in other words, whenever a poison is absorbed in the blood, a diminution takes place immediately in the firmer elements of this fluid; in fact, in some cases, a total destruction of the solid ingredients. In derangements of the liver, spleen and kidneys, this diminution and destruction is very perceptibly marked. Now as soon as this diminution or destruction takes place, the *vis medicatrix naturæ*, that mysterious agent that so admirably controls the equilibrium in health, goes to work with increased energy to reproduce that which disease has destroyed, and we know that if the vitality of our patient is sufficiently active, this regeneration in due time is accomplished. Malarious fevers being induced by poisons from without, acting upon the blood, it is our duty to come up to the assistance of our friend *nature* by neutralizing that poison, and by keeping open, moderately, the secretory and excretory systems, so that all morbid matters may be thrown off, at the same time assisting the same agent in its *formative* process, in bringing back the constituents of the blood to its normal standard. Hence, the very appropriate use of *alkaline* agents in fevers, complicated with or attendant upon inflammations, when there is a constant tendency to consolidation or aggregation of the solid constituents of the blood—saline substances keeping them separate and detached as they exist normally. This treatment I find peculiarly adapted to the first or forming stage of fevers of a typhoid tendency, in preference to the more active one laid down in the majority of our text books. I cannot resist the conclusion, that we very often, in treating our fever patients, entirely overlook the first causes and effects, and rely for the most part upon success in combating the general symptoms as they are developed in the course of the disease. We are more capable of observing the workings of disease before our eyes than any text book can do, yet how many of us are there, who *pin* all our faith to this author or that, pursuing our vocation from day to day, *fac similes* in treatment, yet unmindful of glaring truths taught us in the "Institutes of Medicine," the keystone upon which the superstructure of all our faith should be reared.

We cannot call that system of medicine a *science* that requires us to administer this agent or that, to do this or do that to all patients, because perchance there may be a similitude in the *phenomena* of disease, under which all are laboring. Want of attention, want of knowledge, and want of study, are more to be deplored with us than a want of experience, for the latter is only a question of time, whereas the former becomes farther from us each day of our lives, especially if we place too much reliance upon our experience.

Some one whose name I do not now recollect, has said "that fever is nature's healthy process," necessary to preserve life under circumstances when adverse causes are at work upon the system, fever of itself being no disease, but a natural phenomenon essential to health. The same author goes on to say to the effect that if there were no such thing as *fever*, the world would soon become depopulated, thus proving the healthy tendency of all fevers.

In treating fevers generally, the present age is much in advance of the one just preceding, as is proven by the fact of a diminution in the percentage of mortality; but there are many practitioners who call themselves "regulars" (not to mention any others, and it has not been long since I was an eye-witness to a case I now have in mind), who treat a fever patient very much as one would clean out a stove pipe or a temple of Cloacina's. If nature had no agency whatever in producing health from disease in man, and our patient as many lives as the feline race is said to have, such a course of treatment might be deemed justifiable. Such men have done more to bring a reproach upon the profession than all other causes put together. We seldom if ever find such men members of any medical union for professional advancement and improvement, too old and experienced to apprehend truth revealed to us every day, their libraries and tables minus the scientific treatises and journals of the day, two or three text books partially committed to memory, two bottles labeled hydrar. sub-chloridum and quiniæ sulph., complete their stock in trade. Yet these men are "regulars," practice in the same manner, and use the same medicines the faculty do. Let every other patient be ptyalized under their treatment, and as soon as the tumefaction of the lingual organ subsides, the cry is raised "down with the calomel doctors." Reproach is brought of course on all calomel doctors without discrimination, and homœopathy or some other pathy reaps the benefits.

But it may be said that there is a danger of running into the opposite extreme. This is very true. Extremes have and will always occur while there is so much indiscriminate license given to the practice of medicine; but with those to whom advantages have been given by long and tedious study, to fathom the depths of medical learning in all its ramifications, who are the patient observers of the workings of nature in disease, and who can follow after her in her obscure retreats by the *lights* science has revealed, surely there is less danger of being carried away by any *ignis fatuus* that may perchance be passing by. I will close my remarks by simply stating that in treating fevers generally, we should carefully watch *nature* ourselves, for we are more capable of observing her as she presents herself in disease before our eyes than any book can for us as a general rule;—then an accurate knowledge of the rules of *diet*, as well as those of general medication, is indispensably necessary to a successful practitioner of medicine. Too premature and too much withdrawal of nourishment when nature demands it to perform her task, meddlesome interference and untimely administration of medicaments, should never come up to us in judgment, when quietly reviewing some unsuccessful case at the close of a fatiguing day of professional duties, when the consciousness of a well directed medication should be pleasant and cheering. Let us study *nature* in health and disease more, and routine less, or unite them both as much as we please, but let us not pass by our best friend unnoticed, but recognize her in every aspect, treat with her as circumstances may advise, and she will less frequently turn her back upon us.

